# Epidemiology of sepsis in intensive care units in Turkey: a multicenter, point-prevalence study

**DOI:** 10.1186/s13054-018-2013-1

**Published:** 2018-04-16

**Authors:** Nur Baykara, Halis Akalın, Mustafa Kemal Arslantaş, Volkan Hancı, Çiğdem Çağlayan, Ferda Kahveci, Kubilay Demirağ, Canan Baydemir, Necmettin Ünal, Sepsis Study Group, Pınar Özdemir, Hülya Ulusoy, Dilek Memiş, Cavidan Arar, Elif Yılmaz, Melek Çelik, İsmail Cinel, Sibel Temur, Mehmet Akif Yaşar, Hamdiye Tutan Çulha, Canan Balcı, Sultan San, Asena Salbes, Elçin Özkan, Onur Palabıyık, Jale Çelik, Alper Yosunkaya, Faruk Çiçekçi, Işıl Özkoçak, İsmail Katı, Hülya Başar, Sema Turan, Süheyla Ünver, Ünase Büyükkoçak, Aynur Akın, Sinan Gürsoy, Demet Tok, Simay Serin, Seda Dursun Kıter, Hakan Doğan, Abdülkadir Oruç, Onur Balaban, Dilara Tüfek, İbrahim Kurt, Murat Bilgi, Verda Tuna, Ayşegül Özkök, Ahmet Şen, Nilay Taş, Hilal Ayoğlu, Berna Kaya Uğur, Sedat Kaya, Mustafa Kahraman, Zahide Doğanay, Gökalp Soykam, Işıl Köse, Birgül Yelken, Osman Ekinci, Gönül Ölmez Kavak, Uğur Göktaş, Türkan Togal, Nazım Doğan, Murat Gündüz, Selim Turhanoğlu, Atilla Ramazanoğlu, Füsun Eroğlu, Şaban Yalçın, Hatice Yağmurdur, Atilla Tutak, Ali Aydın Altunkan, Ufuk Kuyrukluyıldız, Gülsüm Oya Hergünsel, Kerem Erkalp, Necati Gökmen, Murat Aksun, Erkan Sayan, Onur Özlü, Yavuz Demiraran, Evren Şentürk, Işın Gençay, Hafize Öksüz, Nihan Yapıcı, Araş Pirat, Tuğhan Utku, Ender Gedik, Orhan Behret, Kadir İdin, Hayrettin Daşkaya, Filiz Banu Ethemoğlu, Ali Alagöz, Seval İzdeş, Mustafa Kemal Bayar, Nermin Kelebek Girgin, Lale Karabıyık, Uğur Koca, Nurcan Çubukçu, Fatma Ülger, Şeyda Efsun Özgünay, Perihan Ergin Özcan, Başar Erdivanlı, Şenay Göksu Tomruk, Abbas Köse, Rafi Doğan, Güldem Turan, Osman Arpaz, Aliye Esmaoğlu, Musa Salmanoğlu, Özgür Canoler, Nedim Çekmen, Canan Kandaz, Gülşen Bulut Kaya, Melis Türker, İlknur Şeker, Aysu Hayriye Tezcan, Halil İbrahim Uzunlar, Mehmet Akif Yazar, Reyhan Arslantaş, Nalan Adıgüzel, Murat Aksun, Okay Öztürk, Zakir Arslan, Özgür Özmen, Günseli Orhun, Evren Büyükfırat, Oya Çimen Budur, Mehmet Erdem Akçay, Asu Özgültekin, Ertuğrul Kılıç, Makbule Elif Yılmaz, Ömür İlban, Feyza Kargın, Murat Sayın, Feyza Kargın, Resul Altuntaş, Hakan Doğan, Osman Ekinci, Berna Çalışkan, Ayşen Erdoğan, Volkan Alparslan, Murat Tekin

**Affiliations:** 1 Department of Anesthesiology, Division of Critical Care, School of Medicine, Kocaeli University, Kocaeli, Turkey; 2 Department of Infectious Disease, School of Medicine, Uludağ University, Bursa, Turkey; 3 Department of Anesthesiology, Division of Critical Care, School of Medicine, Marmara University, Istanbul, Turkey; 4 Department of Anesthesiology, Division of Critical Care, School of Medicine, Dokuz Eylül University, Izmir, Turkey; 5 Department of Public Health, School of Medicine, Kocaeli University, Kocaeli, Turkey; 6 Department of Anesthesiology, Division of Critical Care, School of Medicine, Uludağ University, Bursa, Turkey; 7 Department of Anesthesiology, Division of Critical Care, School of Medicine, Ege University, İzmir, Turkey; 8 Department of Biostatistics and Medical informatics, School of Medicine, Kocaeli, Turkey; 9 Department of Anesthesiology, Division of Critical Care, School of Medicine, Ankara University, Ankara, Turkey

**Keywords:** Intensive care, Sepsis, Carbapenem resistance, Point prevalence, Turkey

## Abstract

**Background:**

The prevalence and mortality of sepsis are largely unknown in Turkey, a country with high antibiotic resistance. A national, multicenter, point-prevalence study was conducted to determine the prevalence, causative microorganisms, and outcome of sepsis in intensive care units (ICUs) in Turkey.

**Methods:**

A total of 132 ICUs from 94 hospitals participated. All patients (aged > 18 years) present at the participating ICUs or admitted for any duration within a 24-h period (08:00 on January 27, 2016 to 08:00 on January 28, 2016) were included. The presence of systemic inflammatory response syndrome (SIRS), severe sepsis, and septic shock were assessed and documented based on the consensus criteria of the American College of Chest Physicians and Society of Critical Care Medicine (SEPSIS-I) in infected patients. Patients with septic shock were also assessed using the SEPSIS-III definitions. Data regarding demographics, illness severity, comorbidities, microbiology, therapies, length of stay, and outcomes (dead/alive during 30 days) were recorded.

**Results:**

Of the 1499 patients included in the analysis, 237 (15.8%) had infection without SIRS, 163 (10.8%) had infection with SIRS, 260 (17.3%) had severe sepsis without shock, and 203 (13.5%) had septic shock. The mortality rates were higher in patients with severe sepsis (55.7%) and septic shock (70.4%) than those with infection alone (24.8%) and infection + SIRS (31.2%) (*p* < 0.001). According to SEPSIS-III, 104 (6.9%) patients had septic shock (mortality rate, 75.9%). The respiratory system (71.6%) was the most common site of infection, and *Acinetobacter* spp. (33.7%) were the most common isolated pathogen. Approximately, 74.9%, 39.1%, and 26.5% of *Acinetobacter, Klebsiella*, and *Pseudomonas* spp. isolates, respectively, were carbapenem-resistant, which was not associated with a higher mortality risk. Age, acute physiology and chronic health evaluation II score at ICU admission, sequential organ failure assessment score on study day, solid organ malignancy, presence of severe sepsis or shock, *Candida spp*. infection, renal replacement treatment, and a nurse-to-patient ratio of 1:4 (compared with a nurse-to-patient ratio of 1:2) were independent predictors of mortality in infected patients.

**Conclusions:**

A high prevalence of sepsis and an unacceptably high mortality rate were observed in Turkish ICUs. Although the prevalence of carbapenem resistance was high in Turkish ICUs, it was not associated with a higher risk for mortality.

**Trial registration:**

ClinicalTrials.gov ID NCT03249246. Date: August 15, 2017. Retrospectively registered.

**Electronic supplementary material:**

The online version of this article (10.1186/s13054-018-2013-1) contains supplementary material, which is available to authorized users.

## Background

Sepsis is a global healthcare problem affecting millions of individuals [1–6]. An increase in the incidence of sepsis in developed countries has been shown in previous studies [1–3]. The incidence rate of sepsis was found to be 535 cases per 100,000 person-years, which is expected to increase in the United States (US) [7]. In a recent meta-analysis of 27 studies from 7 high-income countries, it was estimated that 31.5 million sepsis and 19.4 million severe sepsis cases occur annually worldwide [1]. This trend will likely continue due to the aging population with more chronic illnesses in developed countries, and the increased use of invasive procedures, immunosuppressive therapies, chemotherapies, and transplantations.

Although the sepsis mortality rate has declined in the last two decades due to advances in supportive care, as shown by recent reports from Western countries, the mortality rate is still unacceptably high and survival is frequently associated with long-term morbidity [8–11]. The mortality rate of sepsis (defined by ICD-9-CM) decreased between 1979 and 2000 in the US [10]. Similarly, the hospital mortality of severe sepsis decreased from 35.0% to 18.4% between 2000 and 2012 in Australia and New Zealand [11]. The mortality rate of sepsis might be different among countries and continents due to differences in the provision of intensive care facilities and treatments. According to two multicenter investigations, the hospital mortality rate of severe sepsis was still high (48.7% and 33.5%) in China, which is the fastest-growing country of the world and constitutes a fifth of the world population [12, 13]. The hospital mortality rate of severe sepsis was also found to be high (44.5%) in Asia according to a study performed in 150 intensive care units (ICUs) from 16 countries in 2011 [14].

Currently, there are a limited number of large epidemiologic investigations regarding sepsis in middle- and low-income countries, where almost two-thirds of the world’s population lives. The prevalence and mortality rate of sepsis in Turkey is largely unknown. Turkey is a middle-income country and is among the countries with a high level of antibiotic resistance [15]. It is unclear whether antibiotic resistance alone increases the mortality rate. In previous studies, it has been reported that infections caused by antibiotic-resistant bacteria were associated with a higher mortality ratio, longer ICU stays, and hospitalization costs [16, 17]. However, some other researchers claimed that drug resistance status alone was not a significant predictor of mortality if all patients received the appropriate initial antibiotic treatment [18, 19].

This national, multicenter, point prevalence study was conducted by the Turkish Society of Intensive Care Medicine, Sepsis Study Group to determine the prevalence, causative microorganisms, and mortality rate of sepsis in Turkish ICUs.

## Methods

### Study design, setting, and assembly of cohort

A nationwide, 1-day, point prevalence study was conducted on January 27, 2016. The study was announced on the Turkish Society of Intensive Care Medicine website, which included the study protocol, and the ICU directors (excluding cardiovascular surgery, coronary, and pediatric ICUs) were invited to participate in the study. The other method for recruiting the participating institutions included e-mail invitation to members of the Turkish Society of Intensive Care Medicine.

A total of 132 ICUs from 94 hospitals in Turkey agreed to participate in the study. The Kocaeli University Ethics Committee and Review Board served as the central ethics committee and approved the study for all participating centers (KOÜKAEK 2016/2). A copy of the ethics approval was sent to the study researchers before study commencement. Informed consent was obtained from all participants or their legally acceptable representatives.

All patients (> 18 years old) who were present at the participating ICUs or were admitted for any length of time during a 24-h period between 08:00 on January 27, 2016 and 08:00 on January 28, 2016 were included in the study.

### Data collection: demographic variables and comorbidities

The demographic information, date of ICU admission, admission source, primary diagnosis, comorbidities, acute physiology and chronic health evaluation (APACHE II) score at admission, and sequential organ failure assessment (SOFA) score on study day were recorded for all patients. The presence of the following comorbid conditions was noted: solid organ malignancy, insulin-dependent diabetes mellitus, cerebrovascular accident, New York Heart Association functional class III-IV heart failure, chronic obstructive, restrictive, or vascular pulmonary disease, chronic liver disease, chronic renal failure (patients who are undergoing dialysis or a baseline serum creatinine ≥2 mg.dL^−1^), and immunosuppression [neutropenia (< 500 mm^−3^), hematologic malignancy, splenectomy, human immunodeficiency virus infection (CD_4_ < 200 mm^−3^), chemotherapy-radiation therapy (during the 6 months prior to ICU admission), and corticosteroid treatment in the 6 months before ICU admission (at least 2 weeks > 40 mg.d^−1^ prednisolone, > 160 mg.d^−1^ hydrocortisone, > 32 mg.d^−1^ methylprednisolone, or > 6 mg.d^−1^ dexamethasone) and post-transplantation period].

### Data collection: infection and sepsis

Patients with a confirmed or presumed infection during the 24-h study period were screened for the presence of systemic inflammatory response syndrome (SIRS), severe sepsis, and septic shock based on the modified consensus criteria of the American College of Chest Physicians and Society of Critical Care Medicine (ACCP/SCCM) [20]. The bedside clinicians decided whether the patient had a documented or presumed infection, according to the definitions of the International Sepsis Forum [21]. An extended documentation included the therapeutic intervention, culture and antibiotic susceptibility testing results, types of antimicrobial agent administered, presence of polymicrobial infection or multiple infection, length of ICU stay, and survival status after 1 month in patients with an infection. The survival status of patients who were discharged from the hospital within 30 days of the study day was obtained by follow-up phone calls, either to patients or their families.

Infections present upon admission or developed within 48 h of hospital admission were considered community-acquired. Infections occurring > 48 h after hospital admission were defined as hospital-acquired. Infections that developed at least 48 h after admission into the ICU were defined as ICU-acquired. A polymicrobial infection was defined as isolation of ≥ 2 different microorganisms from the same site of infection. Multiple infection was defined as the presence of ≥ 2 infections simultaneously in the same patient.

SIRS was defined as the occurrence of ≥ 2 of the following criteria: white cell count of > 12,000 cells.mm^−3^ or < 4000 cells.mm^−3^ or > 10% immature forms; heart rate of > 90 beats.min^−1^; temperature > 38 °C or < 36 °C; and respiratory rate > 20 per.min^−1^ or a partial pressure of carbon dioxide < 32 mmHg during spontaneous breathing or the need for mechanical ventilation. In accordance with definitions of the ACCP/SCCM Consensus Conference Committee (SEPSIS-1), sepsis was defined as the presence (documented or presumed) of infection with SIRS [20]. Severe sepsis was defined as sepsis plus at least one sepsis-induced organ dysfunction, which was defined as follows: (a) acute encephalopathy: acute deterioration of neurologic condition (inattention, stupor, delirium, seizures, and coma), (b) hematological dysfunction: platelet count < 100,000 μL^−1^, (c) respiratory dysfunction: PaO_2_/fraction of inspired oxygen < 200 if lungs are the site of infection or < 300 if lungs were not the infection site, (d) renal dysfunction: urinary output < 0.5 mL.kg.h^−1^ for at least 2 h despite adequate volume resuscitation or serum creatinine > 2 mg.dL^−1^ not attributable to chronic renal failure or > 50% increase from known baseline, (e) lactic acidosis: plasma lactate level > 2 mmol.L^−1^, and (f) liver dysfunction: bilirubin > 2 mg.dL^−1^ or international normalized ratio > 1.5 in the absence of anticoagulant agents. Septic shock was defined as severe sepsis associated with refractory hypotension; despite at least 2 h of adequate volume resuscitation, a systolic blood pressure (SBP) < 90 mmHg or a reduction of ≥40 mmHg from baseline level or a mean arterial pressure < 70 mmHg in the absence of other causes of hypotension or the need for vasopressors to maintain SBP ≥ 90 mmHg.

This study was performed before the publication of the new sepsis definitions (SEPSIS-III) [22]. However, we determined patients who were in septic shock according to the SEPSIS-III definitions using our collected data. The clinical criteria to identify septic shock according to SEPSIS-III definitions were as follows: the need of vasopressor therapy to sustain a mean arterial pressure ≥ 65 mmHg and serum lactate levels > 2 mmol∙L^−1^ persisting after adequate fluid replacement [22].

### Data source

A detailed study protocol containing definitions for various items and instructions for data collection were sent to the study participants by e-mail. The principal investigators were easily accessible to all participants for all queries during data collection. Both bedside observation charts (demographic and clinical data) and electronic patient records (laboratory data regarding microbiology and antimicrobial resistance) could be used to capture data. All data were initially recorded using standardized paper-based forms. ICU and patient details were recorded in two different forms. The first form (Form A) was for the hospital and ICU details, such as hospital type, hospital and ICU bed counts, ICU type, total number of patients in the ICU on study day, nurse-to-patient ratios, number of ICU beds, and the percentage of ICU bed occupancy. The second form (Form B) was the case report form (CRF). The investigators also filled electronic CRFs on a specialized website (http://www.yogunbakim.org.tr/tybdaa/). All records were collected and evaluated by the principal investigators in the study center. After reviewing the data, the principal investigator asked the other investigators to fill out missing values.

### Statistical analysis

All statistical analyses, except for the binary logistic regression analyses, were performed using SPSS version 20.0 (SPSS Inc, Chicago, IL, USA). Stata/MP 13.0 (StataCorp, College Station, TX, USA) for Windows was used for binary logistic regression analyses. Student’s *t* test and one-way analysis of variance with Tukey’s post hoc test were used to compare normally distributed continuous variables. The Kruskal-Wallis test with Dunn’s post hoc test and Mann-Whitney *U* test were used to compare non-normally distributed continuous variables. According to the expected and observed frequency, Pearson’s chi-square, Yates’ chi-square, or Fisher’s exact test was used for contingency tables. Pearson’s chi-square or Monte Carlo simulation was applied for contingency tables that were larger than 2×2 according to the expected and observed frequency.

Two separate analyses were conducted to assess the relationship between carbapenem resistance and mortality. First, mortality rates were compared with the chi-squared test between patients infected with carbapenem-resistant (CR) and carbapenem-sensitive (CS) strains. For the second analysis, patients infected with *Acinetobacter*, *Klebsiella,* or *Pseudomonas* spp. were pooled. We initially compared demographics, comorbidities, APACHE II at admission day, sepsis severity, site of infection, and the length of stay prior to study day between patients who died and lived at 30 days. Variables with a *p* value of < 0.2 on univariate analysis were included in a multiple binary logistic regression analysis. The CR infection, polymicrobial infection, and multiple infection variables were included in the model regardless of statistical significance. The other variables included in the multiple logistic regression analysis were infection with *Acinetobacter, Klebsiella,* or *Pseudomonas* spp. Multiple logistic regression, with backward elimination method, was conducted to assess the predictors of mortality. A jackknifing resampling technique was used for multiple logistic regression to assess biases within the dataset. Multicollinearity was evaluated, with a variance inflation factor of > 10 as an exclusion criteria. Box-Tidwell test was used to check the assumption of linearity of continuous predictors with logit-mortality. Significance levels for this test for age, APACHE II, SOFA, and the length of stay prior to study day were 0.938, 0.082, 0.711, and 0.074 respectively. Variables with a *p* value of < 0.05 were retained in the final model. There was a limited number of patients infected with methicillin-sensitive/resistant *Staphylococcus aureus*; therefore, only chi-squared test was performed to compare the mortality rates between patients infected with methicillin-sensitive/resistant *S. aureus*.

A second multiple logistic regression analysis was performed to ascertain factors affecting mortality in the entire cohort of infected patients. Because of the hierarchical structure of the data, at first, three-level logistic regression analysis was considered to account for the within-cluster correlation of patient outcomes (at 30 days) in the entire cohort of infected patients. Levels were constructed as patients (level-1) nested within ICUs (level-2) and ICUs nested within hospitals (level-3). For all the selected independent variables, we calculated the intra-class correlation coefficients for the hospital (ICCh) and for the ICU level (ICCi) and corresponding design effects. When analyzing the association between mortality and variables, the ICCh ranged from 0.01 to 0.04 and the ICCi ranged from 0.05 to 0.11 (Additional file 1: Table S1). Design effects (i.e., indices of the extent of clustering effects) ranged from 1.06 to 1.68 in all cases (Additional file 1: Table S1). Since design effects were lower than the critical value of two in all cases [23], single-level multiple logistic regression, with backward elimination method, was conducted to assess the predictors of mortality. A jackknifing resampling technique was used for multiple logistic regression to assess biases within the dataset. Variables considered for the multivariate modelling in the entire cohort included demographic data, comorbidities, admission category, severity scores (APACHE 2 score on admission, SOFA score on study day), clinical condition, presence of organ dysfunction and lactic acidosis, site of infection, type of microorganism, presence of polymicrobial infection and multiple infection, types of hospital and ICU, size of hospital, nurse to patient ratio, and therapies used. Variables with a *p* value of < 0.2 on univariate analysis were included into multiple binary logistic regression analysis. Polymicrobial infection and multiple infection variables were included in the model regardless of statistical significance. Multicollinearity was evaluated, with a variance inflation factor of > 10 as an exclusion criteria. VIF values of selected variables were between 1.03 and 1.50. The Box-Tidwell test was used to check the assumption of linearity in continuous predictors with logit-mortality. Significance levels for this test for age, APACHE-II, and SOFA were all 0.757, 0.981, and 0.321 respectively. A *p* value of < 0.05 was considered significant. Adjusted odds ratios with 95% confidence intervals (CIs) were reported.

## Results

### Hospital and ICU characteristics

ICUs (*n* = 132) from 94 hospitals, which were located in all 7 geographical regions of Turkey, participated in this study. The types of hospitals were as follows: university hospitals (*n* = 52; 55.3%), education and research hospitals (*n* = 24; 25.5%), state hospitals (*n* = 10; 10.6%), and private hospitals (*n* = 8; 8.5%). Approximately 71.2% of ICUs were mixed medical and surgical. In 63.5% of the participating ICUs, the nurse-to-patient ratio was 1:3 (44.6%) or 1:4 (18.9%) (Table 1). The average percentage of ICU bed occupancy was 92.7% ± 11.4% .

**Table 1:** Characteristics of participating centers

Characteristics	All centers (N, %)
Type of hospital	94 (100)
University hospital	52 (55.3)
Education and research hospital^a^	24 (25.5)
State hospital	10 (10.6)
Private hospital	8 (8.5)
Specialty of ICU head	132 (100)
Intensivist	66 (50)
Anesthesiologist	46 (34.1)
Surgeon	8 (6)
Internist	5(3.7)
Neurologist	3 (2.2)
Pulmonologist	3 (2.2)
Interdisciplinary	1 (0.7)
Type of ICU	132 (100)
Mixed medical/surgical	94 (71.2)
Surgical	19 (14.3)
Medical	16 (12.1)
Neurological	3 (2.2)
Nurse-to-patient ratio	132 (100)
1:2	48 (36.3)
1:3	59 (44.6)
1:4	25 (18.9)
The average percent (±SD), and CI 95% of ICU bed occupancy	92.7% ± 11.4% (91.5-93.0)

*ICU* intensive care unit, *SD* standard deviation, *CI* confidence interval

^a^Education and research hospitals are different from university hospitals, because they conduct postgraduate medical education and research. The former only provides specialty training (i.e., doctors who specialize after graduating from a medical faculty), whereas the latter provides specialty and medical training concurrently

**Fig. 1:**
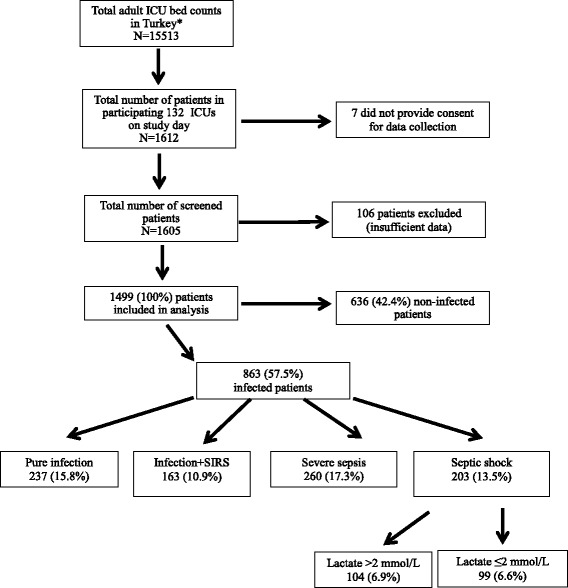
Flow chart of study participants. *ICU* intensive care unit, *SIRS* systemic inflammatory response syndrome.,*Following exclusion of cardiovascular, coronary, and pediatric ICUs

The flowchart of the study is shown in Fig. 1.

### Prevalence and distribution of infection and sepsis

Infection was present in 863 (57.5%) of all patients. According to the 1992 consensus definitions, out of the 1499 patients included in the analysis, 163 (10.9%) had sepsis (confirmed/presumed infection plus SIRS), 260 (17.3%) had severe sepsis without shock, and 203 (13.5%) had septic shock (Fig. 1). According to the SEPSIS-III definitions, 104 (6.9%) patients were classified as having septic shock.

The infection rate was higher in hospitals with a bed capacity < 200 compared to that in hospitals with a higher bed capacity (*p* ≤ 0.05). However; the distribution of infected patients (i.e., pure infection, sepsis, severe sepsis, and septic shock) were not different among the hospital types (*p* = 0.169), and sizes (*p* = 0.068), and ICU types (*p* = 0.120). (Additional file 2: Table S2).

The patient characteristics stratified according to the severity of sepsis and survival status are shown in Table 2. Patients who had septic shock on study day were older than patients with sepsis (*p* = 0.046) and had higher admission APACHE II scores than other infected patients without shock (*p* < 0.05). Patients with severe sepsis or septic shock had higher SOFA scores than patients without severe sepsis on study day (*p* ≤ 0.001), and SOFA scores of patients with septic shock were higher than severe septic patients without shock (Table 2, *p* = 0.001). The most frequent comorbid condition was chronic respiratory failure, followed by cerebrovascular accident and congestive heart failure in infected patients. The rate of severe sepsis (with or without shock) was 71.6% in infected patients who have immunosuppression as a comorbid condition.

**Table 2:** Patient characteristics and mortality ratio stratified according to sepsis severity

	All infected patients *n* = 863	Infection *n* = 237	Infection + SIRS *n* = 163	Severe sepsis without shock *n* = 260	Septic shock (SEPSIS I) *n* = 203	Septic shock^a^(SEPSIS-III) *n* = 104
Age, yrs	69 (55–79)	70 (58–80)	64 (52–78)	69 (50–78)	70 (58–79)^*^	67 (57–76)
Female/male, n	366/497	104/133	68/95	108/152	86/117	46/58
APACHE II score at ICU admission^b^	21 (16–28)	20 (15–24)	18.0 (15–24)	21.5 (17–28)^‡^	25(19–31)^c^	25.5(19.7-31.2)
SOFA score ^b^	7 (5–11)	6 (4–9)	5 (3–8)	8 (6–11)^ƒ^	10 (7–13)^ƒ, †^	11 (8–14)
Comorbidities, n (%)						
Chronic respiratory disease	192 (22.3)	52 (21.9)	39 (23.9)	57 (21.9)	44 (21.7)	24 (23.0)
Cerebrovascular accident	118 (13.6)	34 (14.3)	29 (17.7)	36 (13.8)	19 (9.4)	12 (11.5)
Congestive heart failure	98 (11.3)	21 (8.4)	18 (10.4)	0 (10.0)	29 (17.2)	18 (17.3)
Chronic renal failure	95 (11)	23 (9.7)	18 (11)	28 (10.7)	26 (12.8)	14 (13.5)
Solid organ malignancy	81 (9.3)	17 (7.1)	14 (8.5)	24 (9.2)	26 (12.8)	15 (14.4)
Immunosuppression	67 (7.7)	9 (3.8)	10 (6.1)	22 (8.4)^Ø^	26 (12.8)^#^	21 (20.1)
Liver disease	22 (2.5)	5 (2.1)	1 (0.6)	7 (2.6)	9 (4.4)	7 (6.7)
Alcoholism	17 (1.9)	6 (2.5)	3 (1.8)	3 (1.2)	5 (2.5)	2 (1.9)
Number of acute organ dysfunctions (mean ± SD)		–	–	2.0 ± 1.0	3.7 ± 1.5^&^	4.4 ± 1.3
Organ dysfunction, n (%)						
Respiratory	271 (31.4)	–	–	147 (56.5)	124 (61.1)	74 (71.2)
Renal	223 (25.8)	–	–	104 (40.0)	119 (58.6)^&^	70 (67.3)
Neurologic	171 (19.8)	–	–	70 (26.9)	101 (49.8)^&^	56 (53.8)
Liver	133 (15.4)	–	–	70 (26.9)	63 (31.0)	38 (36.5)
Hematologic	118 (13.6)	–	–	64 (24.6)	54 (26.6)	36 (34.6)
Lactic acidosis (> 2 mmol∙L^−1^), n (%)	174 (20.2)	–	–	83 (31.9)	91 (44.8)^€^	104 (100)
Therapies, n (%)						
RRT	152 (17.6)	21 (8.9)	22 (13.5)	53 (21.5)	56 (26.1)	28 (26.9)
MV	715 (82.9)	176 (74.3)	123 (75.5)	227 (87.3)	189 (93.1)	94 (90.4)
Length of stay, day^e^	27 (13–45)	30 (13–47)	27 (13–48)	30(14.2–45.7)	22 (11.2-36.5^)¢^	17 (10–30)
Mortality, n (%)	401 (46.1)	61 (24.8)	52 (31.2)	145 (55.7)^ƒ^	143 (70.4)^ƒ, †^	79 (75.9)

Data are presented as median (25th–75th percentiles), if not otherwise specified

*SIRS* systemic inflammatory response syndrome, *APACHE II* acute physiology and chronic health evaluation, *ICU* intensive care unit, *SOFA* sequential organ failure assessment, *SD* standard deviation, *RRT* renal replacement therapy, *MV* mechanical ventilation

^a^Patients with septic shock based on SEPSIS-III definitions are covered in the septic shock group based on the SEPSIS-1 definition; data presented in this column were not included in any statistically analysis

^b^8 and ^e^56 missing values

*p = 0.046, compared with infection + SIRS group

^**c**^*p* ≤ 0.05, compared with infection, infection + SIRS, and severe sepsis groups

^‡^*p* = 0.042, compared with infection + SIRS groups

^ƒ^*p* ≤ 0.001, compared with infection and infection + SIRS groups

^†^*p* = 0.001, compared with severe sepsis groups

^Ø^*p* = 0.031, compared with infection group

^¢^*p* = 0.027, compared with infection group

^#^*p* ≤ 0.05, compared with infection and infection + SIRS groups

^&^*p* ≤ 0.001, compared with severe sepsis groups

^€^*p* = 0.005, compared with severe sepsis groups

Approximately 32.8% of all infected patients had community-acquired infections, whereas 54.4% of infected patients had nosocomial infections in the present study (Table 3). The most common site of infection was the respiratory system (71.6%), followed by the bloodstream (8.9%) and urinary system (7.8%) in infected patients. Except for skin/soft tissue infections, the distribution of infections was similar among patients with different clinical conditions, including infection alone, infection plus SIRS, severe sepsis, and septic shock. None of the patients with skin/soft tissue infections had septic shock on study day (Table 3).

**Table 3:** Origin and type of infection in infected patients

	All infected patients n = 863	Infection n = 237	Infection + SIRS n = 163	Severe sepsis without shock n = 260	Septic shock (SEPSIS I) n = 203	Septic shock^a^(SEPSIS-III) n = 104
Origin of infection, n (%)						
Community-acquired	285 (32.8)	85 (35.8)	52 (31.9)	86 (33)	62 (30.5)	30 (28.8)
Hospital-acquired	259 (30)	59 (24.8)	52 (31.9)	75 (28.8)	73 (35.9)	38 (36.5)
ICU-acquired	211 (24.4)	62 (26.1)	44 (26.9)	64 (24.6)	41 (20.1)	21 (20.1)
Unknown	108 (12.5)	31 (13.0)	15 (9.2)	35 (13.4)	27 (13.3)	15 (14.4)
Type of infection^b^, n (%)						
Respiratory	618 (71.6)	158 (66.6)	118 (72.3)	188 (72.3)	154 (75.9)	85 (81.7)
Bloodstream	77 (8.9)	28 (11.8)	15 (9.2)	22 (8.5)	12 (5.9)	9 (8.6)
Renal/urinary	67 (7.8)	21 (8.8)	12 (7.4)	19 (7.3)	15 (7.3)	7 (6.7)
Catheter-related	56 (6.5)	17 (7.1)	8 (4.9)	18 (6.9)	13 (6.4)	6 (5.7)
Intra-abdominal	49 (5.6)	10 (4.2)	9 (5.5)	12 (4.6)	18 (8.8)	13 (12.5)
Surgical	32 (3.7)	6 (2.5)	5 (3.0)	9 (3.4)	12 (5.9)	3 (2.8)
Skin/soft tissue	24 (2.7)	6 (2.5)	8 (4.9)	10 (3.8)	0 (0)^*^	0 (0)
Others	22 (2.5)	2 (0.8)	6 (3.7)	9 (3.4)	5 (2.5)	3 (2.9)

*SIRS* systemic inflammatory response syndrome, ICU intensive care unit

^a^Patients with septic shock based on SEPSIS-III definitions are covered in the septic shock group based on the SEPSIS-1 definition and data presented in this column were not included in any statistically analysis

^b^Percentages do not necessarily equal 100 because patients may have had > 1 type of infection

^*^p < 0.05, compared with infection, infection + SIRS, and severe sepsis groups

Of the 503 (58.3%) of infected patients had positive microbial isolates. 78.7 of the positive isolates were gram negative, 15.5% were gram positive, 4.9% were fungi, and 0.7% were viruses (Table 4). *Acinetobacter* spp. were the most common isolated pathogen (33.7%), followed by *Pseudomonas* spp. (16.4%) and *Klebsiella* spp. (16.0%; Table 4). About 74.8% of *Acinetobacter* spp. isolates, 39.0% of *Klebsiella* spp. isolates, and 26.5% of *Pseudomonas* spp. isolates were resistant to carbapenem. It was observed that 2.7% of *Klebsiella* spp. isolates, 2.6% of *Pseudomonas* spp. isolates, and 2.1% of *Acinetobacter* spp. isolates were resistant to colistin. About 57.0% of the gram-positive isolates were *S. aureus*, and 75.4% of the *S. aureus* isolates were identified as methicillin-resistant (MRSA). The distribution of microorganisms isolated from culture-positive-infected patients according to their clinical conditions is shown in Table 4. The proportions of isolated microorganisms were not significantly different among patients with different clinical conditions, including infection alone, infection + SIRS, severe sepsis, and septic shock, when evaluated using Monte Carlo simulation.

**Table 4:** Distribution of microorganisms isolated from culture-positive-infected patients according to clinical condition

	All (n = 863)	Infection (n = 237)	Infection + SIRS (n = 163)	Severe sepsis without shock (n = 260)	Septic shock (n = 203)
Culture-positive-infected patients, n (%)	503 (58.3)	131(55.3)	83 (50.9)	154 (59.2)	135 (66.5)
Isolated microorganisms^a^, n (%)	686(100)	161(100)	126(100)	213(100)	186(100)
Gram negative	540 (78.7)	124 (77)	99 (78.5)	162 (76)	155 (83.3)
*Acinetobacter* spp.	231 (33.7)	56 (34.7)	42 (33.3)	70 (32.8)	63 (33.9)
Carbapenem-resistant	173 (25.2)	37 (22.9)	32 (25.3)	51 (23.9)	53 (28.5)
Colistin-resistant	5 (0.7)	1 (0.6)	1 (0.8)	1(0.5)	2 (1.0)
*Pseudomonas* spp.	113 (16.4)	22 (13.6)	22 (17.4)	39 (18.3)	30 (16.1)
Carbapenem-resistant	30 (4.4)	6 (3.7)	8 (6.3)	10 (4.7)	6 (3.2)
Colistin-resistant	3 (0.4)	0 (0.0)	1 (0.8)	0 (0.0)	2 (1.0)
*Klebsiella* spp.	110 (16)	23 (14.2)	20 (15.8)	26 (12.2)	41 (22.0)
Carbapenem-resistant	43 (6.3)	10 (6.2)	8 (6.3)	7 (3.3)	18 (9.7)
Colistin-resistant	3 (0.4)	0 (0.0)	1 (0.8)	0 (0.0)	2 (1.0)
*Escherichia coli*	37 (5.4)	12 (7.4)	6 (4.7)	8 (3.7)	11 (5.9)
*Serratia* marcescens	12 (1.7)	3 (1.8)	2 (1.5)	6 (2.8)	1 (0.5)
*Proteus spp.*	10 (1.4)	3 (1.8)	0 (0)	6 (2.8)	1 (0.5)
*Enterobacter* spp.	8 (1.1)	2 (1.2)	0 (0)	4 (1.8)	2 (1.0)
Others	19 (2.7)	3 (1.8)	7 (5.5)	3 (1.4)	6 (3.2)
Gram positive	107 (15.5)	26 (16.1)	21 (16.6)	41 (19.2)	19 (10.2)
*Staphylococcus aureus*	61 (8.9)	16 (9.9)	13 (10.3)	20 (9.8)	12 (6.4)
*MRSA*	46 (6.7)	13 (8.0)	12 (9.5)	12 (5.6)	9 (4.8)
*Enterococcus* spp.	38 (5.5)	9 (5.6)	8 (6.3)	14 (6.5)	7 (3.7)
*VRE*	3 (0.4)	1 (0.6)	0 (0)	2 (0.9)	0 (0)
Others	8 (1.2)	1 (0.6)	0 (0)	7 (3.3)	0 (0)
Fungi	34 (4.9)				
*Candida* spp.	32 (4.7)	8 (4.9)	6 (4.7)	8 (3.7)	10 (5.4)
*Aspergillus* spp.	2 (0.3)	0 (0)	0 (0)	0 (0)	2 (1.0)
Virus	5 (0.7)				
H1N1	5 (0.7)	3 (1.9)	0 (0)	2 (0.9)	0 (0)

*SIRS* systemic inflammatory response syndrome, *MRSA* methicillin-resistant *Staphylococcus aureus VRE* vancomycin-resistant Enterococcus

^a^Patients may have more than one microorganism isolated

### Therapies

All infected patients were receiving at least one antimicrobial agent on study day. The most frequently administered antimicrobials to patients with infections on study day were as follows: carbapenems (*n* = 322; 37.3%), followed by colistin (*n* = 180; 20.8%), cephalosporins (*n* = 120;13.9%), fluoroquinolones (*n* = 119;13.7%), glycopeptides (*n* = 90; 10.4%), antifungals (*n* = 70; 8.1%), tigecycline (*n* = 65; 7.5%), beta lactam-betalactamase inhibitors (*n* = 62; 7.2 0%), linezolid (*n* = 57; 6.6%), antivirals (*n* = 54; 6.2%), clindamycin (*n* = 36; 4.2%), metronidazole/ornidazole (*n* = 21; 2.4%), aminoglycosides (*n* = 15; 1.7%), and others (*n* = 25; 2.9%).

The monitoring methods and therapies used in patients with severe sepsis (with or without shock) on study day are shown in the Additional file 3: Table S3. Mechanical ventilation (MV) was required in 87.2% of patients with severe sepsis. Inotropic/vasopressor agents, corticosteroids, and renal replacement therapy (RRT) were the other most frequently used life support therapies in patients with severe sepsis (49.0%, 26.7%, and 23.7%, respectively). Although the lactate level was recorded on study day in all infected patients according to the requirements of the study protocol, the lactate levels were measured as a monitoring method in only 79.9% of patients with severe sepsis (Additional file 3: Table S3).

### Organ dysfunction and mortality

Respiratory and renal dysfunction were the most common organ dysfunctions among patients with severe sepsis and septic shock (Table 2). However, renal dysfunction (*p* ≤ 0.001), neurologic dysfunction (*p* ≤ 0.001), and lactic acidosis (*p* = 0.005) were significantly more common in septic shock patients than in severe septic patients without shock (Table 2). The mortality rates of severe sepsis and septic shock (55.7% and 70.4%, respectively) were significantly higher than that in patients with infection alone and infection plus SIRS (24.8% and 31.3%, respectively) (*p* ≤ 0.001; Table 2).

Septic shock was associated with a higher number of organ dysfunctions (4.4 ± 1.3 and 3.7 ± 1.5, respectively) and mortality rate (75.9% and 70.4%, respectively) when septic shock was defined according to SEPSIS-III definition compared to the SEPSIS-I definitions (Table 2). The sensitivity and specificity of septic shock for mortality rate were higher (80.4% and 94%, respectively) when the SEPSIS-III definitions were used in comparison to the SEPSIS-I definitions (64.2% and 87.4%, respectively).

### Antibiotic resistance and mortality

**Fig. 2:**
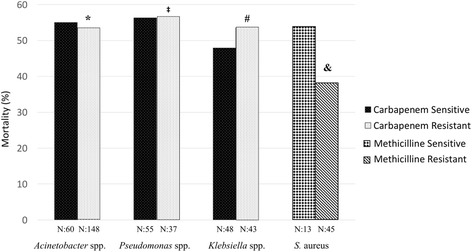
Antibiotic susceptibility pattern of microorganisms and survival status at 30 days. Mortality ratio was not different among antibiotic-sensitive and -resistant strains when evaluated by chi-squared test. *p = 0.832, compared with carbapenem-sensitive *Acinetobacter* spp. ^‡^*p* = 0.970, compared with carbapenem-sensitive *Pseudomonas* spp. ^#^*p* = 0.595, compared with carbapenem-sensitive *Klebsiella* spp. ^&^*p* = 0.474, compared with methicillin-sensitive *S. aureus*

As shown in Fig. 2, the rate of mortality in those with MRSA infections was not statistically different from that of methicillin-sensitive *S. aureus* infections. The mortality rate was also not different between CR and CS strains (Fig. 2).

Patients infected with *Acinetobacter* spp., *Klebsiella* spp., or *Pseudomonas* spp. were pooled. A binary logistic regression analysis was performed to identify risk factors for mortality among patients infected with *Acinetobacter* spp., *Klebsiella* spp., or *Pseudomonas* spp. Variables with a *p* value < 0.2 on univariate analysis (Additional file 4, Table S4) were incorporated into the multiple binary logistic regression model The CR infection, polymicrobial infection, and multiple infection variables were included in the model regardless of statistical significance. The other variables included in the binary logistic regression analysis were infection with *Acinetobacter, Klebsiella,* or *Pseudomonas* spp. Based on the multiple binary logistic regression analysis, it was confirmed that carbapenem resistance was not an independent risk factor for mortality (*p* = 0.587) after adjusting for other variables in the model. However, age, severe sepsis, septic shock, and RRT were independent risk factors for mortality (Table 5). Infection with *Klebsiella* spp. was associated with a lower mortality among patients infected with *Acinetobacter, Klebsiella,* or *Pseudomonas* spp. (Table 5).

**Table 5:** Risk factors for 30-day mortality in patients infected with *Acinetobacter*, *Pseudomonas*, or *Klebsiella* spp

	Odds ratio	95% CI	*p* value
Age, per year increase	1.02	1.012–1.040	< 0.001
Clinical condition			
Infection^a^			
Infection + SIRS	1.983	0.960–4.096	0.064
Severe sepsis without shock	3.072	1.603–5.889	< 0.001
Septic shock	7.587	3.765–15.287	< 0.001
Infections			
*Klebsiella* spp.	0.550	0.316–0.960	0.035
Therapies			
RRT	1.903	1.031–3.513	0.040

Model log-likelihood: -208.427; F: 8.55; *p* ≤ 0.001. Hosmer-Lemeshow X^2^ = 10.2; *p* = 0.247

*CI* confidence interval, *SIRS* systemic inflammatory response syndrome, *RRT* renal replacement therapy

^a^Reference group

### Factors associated with mortality in the entire cohort of infected patients

Comparative analyses of the variables in relation to mortality in all infected patients are shown in the Additional file 5: Table S5. By univariate analysis, age, APACHE-II score at admission, SOFA score on study day, and the presence of comorbid conditions, including congestive heart failure, chronic renal failure, solid organ malignancy, chronic liver disease, and immunosuppression, were associated with mortality. Based on the univariate analysis, the presence of any acute organ dysfunctions (i.e., respiratory, renal, hepatic, neurologic, and hematologic dysfunction), hyperlactatemia, the severity of sepsis, *Acinetobacter* spp. infection, *Pseudomonas* spp. infection, *Candida* spp. infection, polymicrobial infection, RRT, and MV were also associated with mortality (Additional file 5: Table S5). Variables with a *p* value < 0.2 on univariate analysis were incorporated into the multiple logistic regression model. In the multiple logistic regression analysis, APACHE-II scores at ICU admission, SOFA scores on study day, older age, solid organ malignancy, the presence of severe sepsis, or septic shock, *Candida* spp. infection, RRT, and a nurse-to-patient ratio of 1:4 (compared with a nurse-to-patient ratio of 1:2) were independent predictors of mortality in the entire group of infected patients (Table 6).

**Table 6:** Risk factors for 30-day mortality in all infected patients

	Odds ratio	95% CI	*p* value
Age, per year increase	1.02	1.012–1.031	< 0.001
APACHE II score at admission, per point increase	1.02	1.006-1.036	0.005
SOFA score on study day, per point increase	1.115	1.067–1.165	< 0.001
Comorbid condition			
Solid organ malignancy	1.924	1.081–3.425	0.026
Clinical condition			
Infection^a^			
Infection + SIRS	1.617	0.986–2.651	0.057
Severe sepsis	4.659	2.875–7.548	< 0.001
Septic shock	3.326	2.152–5.141	< 0.001
Infections			
Candida spp.	3.526	1.437–8.653	0.006
Therapies			
RRT	2.675	1.702–4.206	< 0.001
Nurse to patient ratio			
1:2^a^			
1:3	1.43	0.977–2.014	0.066
1:4	1.958	1.259–3.043	0.003

Model log-likelihood: -474.943; F: 15.15; *p* ≤ 0.001. Hosmer-Lemeshow X^2^ = 10.3; *p* = 0.245

*CI* confidence interval, *APACHE II* acute physiology and chronic health evaluation, *SOFA* sequential organ failure assessment, *SIRS* systemic inflammatory response syndrome, *RRT* renal replacement therapy

^a^Reference group

## Discussion

In this large-scale, multicenter study, we observed a high incidence of infection and sepsis in Turkish ICUs. Moreover, the mortality rate of severe sepsis and septic shock was unacceptably high (55.7% and 70.4%, respectively). Although the prevalence of carbapenem resistance was high in Turkish ICUs, it was not associated with a higher risk for mortality. However, age, APACHE-II score at ICU admission, SOFA score on study day, solid organ malignancy, the presence of severe sepsis or septic shock, *Candida* spp. infection, RRT, and a nurse-to-patient ratio of 1:4 were found to be predictors of mortality.

According to our study, the infection rate was 57.5% and about 54.4% of the infections were nosocomial in Turkish ICUs. In a previous, multicenter, point prevalence study performed in September 2004, Esen et al. reported that 48.7% of patients were infected in Turkish ICUs and, similar to our findings, the most frequent site of infection was the respiratory system, followed by bloodstream and urinary tract infections [24]. In the international study of the prevalence and outcomes of infection in intensive care units (EPIC II), the prevalence of ICU infections was higher in countries that allocated a lower percentage of their gross domestic product on healthcare [25]. Many factors, including infection control practices, educational strategies, national antibiotic and public health policy can affect infection rates [25]. Although healthcare spending in Turkey has increased rapidly since 2002, Turkey remains the country with the lowest health expenditure in the OECD [26]. Turkey also has fewer critical human resources for healthcare compared to developed countries. A challenge for infection control in healthcare facilities in Turkey is a low nurse-to-patient ratio [27]. One of the most important findings of this study was that the low nurse-to-patient ratio in ICUs was an independent risk factor for mortality in infected patients. Some researchers observed no association [28, 29] while some found significant associations [30, 31] between the nurse-to-patient ratio and mortality rate in previous studies. The optimal nurse-to-patient ratios have not yet been determined based on scientific evidence. In the present study, the odds of death was increased by 1.95 (95% CI, 1.2–3.0) when the nurse-to-patient ratio was 1:4 instead of 1:2 in infected patients. In a recent multicenter, longitudinal study, the risk of death increased by 3.5 (95% CI, 1.3–9.1) in the ICU when the patient to nurse ratio was > 2.5 [30]. Further studies are needed to demonstrate the optimal nurse-to-patient ratio in the ICU.

### The prevalence of sepsis, severe sepsis and septic shock

In the present study, the prevalence of sepsis, severe sepsis, and septic shock was 10.9%, 17.3%, and 13.5%, respectively, in infected patients according to the 1992 definitions (SEPSIS-I). The total prevalence of severe sepsis was high (30.9% with or without shock) in the present study. The prevalence of severe sepsis was found to be 26% and 22% in two single-day, point prevalence studies performed in 2012 and 2013, respectively, in Polish ICUs [32]. According to point prevalence surveys, the prevalence of severe sepsis was 29.6% in Brazilian ICUs on a single day 2015 [33] and 17.9% in German ICUs in 2013 [3].

The percentage of sepsis in point prevalence studies changes according to the availability of ICU beds (34). It was claimed that the high frequency of sepsis in ICUs in some countries, such as Brazil and the United Kingdom (UK), are due to a shortage of ICU beds because only the most seriously ill patients (i.e., those with sepsis and multiorgan failure) can be admitted [34, 35]. Although the ICU bed counts in Turkey can be comparable to that of countries in Western Europe (www.saglikistatistikleri.gov.tr>SIY-2015), there is always a high demand for ICU beds in Turkey, mostly due to a lack of post-ICU care facilities and legal support to limit life-support interventions for terminally ill patients. Therefore, Turkish ICUs are mostly overcrowded and only the sickest patients can be admitted. This situation also causes delay in treatment, preventing the early identification and intervention of sepsis, which may worsen outcomes [36].

### Mortality of severe sepsis

In a recent, international, multicenter, prevalence study on sepsis (IMPRESS study) [37], the average mortality rate of severe sepsis was 28%; however, there were large differences in the mortality rates among North America, South/Central America, and Eastern Europe (24.2%, 36.7%, and 44%, respectively). The 30-day mortality rate of severe sepsis and septic shock was unacceptably high (55.8% and 70%, respectively) in the present study. Although there is no previous multicenter study regarding sepsis in Turkey, a very high ICU mortality rate of 87.3% was reported in a single-center retrospective study from a university hospital in Turkey based on the review of data from 63 sepsis patients from 2002 to 2003 [38]. We did not investigate compliance with sepsis bundles in Turkish ICUs in this study. However, in a previous multicenter study, it was found that physicians who were routinely in charge of sepsis patients in Turkey had poor knowledge of the sepsis bundles [39]. Delayed treatment and poor compliance with sepsis bundles are probably the most important causes of high mortality in patients with severe sepsis in Turkish ICUs. This study was performed in the winter (January 27), which could have contributed to the high frequency and mortality rate of sepsis as shown in previous studies [40].

High mortality rates for sepsis and septic shock, similar to that of Turkey, have been reported in other middle- and low-income countries. According to point prevalence surveys, the overall mortality rate of severe sepsis was 55.7% in Brazilian ICUs, 64.6% in Indian ICUs, and 80% in Pakistan ICUs [33, 41, 42]. According to a single-center cohort study, the mortality ratio of septic shock was 82% in Tunisia [43].

### Carbapenem resistance and mortality

The carbapenem resistance rates in the present study (*Acinetobacter* spp., 74.9%; *Klebsiella* spp., 39.1%; and *Pseudomonas* spp., 26.5%) were higher than that of previous reports from most of European countries and the US [44, 45]. Carbapenem resistance was not found to be an independent risk factor for mortality in the present study, which may be explained in part by the fact that there is widespread use of polymyxins for treating CR infections in Turkey. In the present study, one in every five infected patients received colistin on study day. In some previous studies, more deaths were observed among patients infected with CR Enterobacteriaceae than those with CS Enterobacteriaceae [16, 17]; however, there was no significant difference in mortality rates for patients infected with CR and CS Enterobacteriaceae in some other studies [18, 19]. In the present study, infections were defined according to the definitions of the International Sepsis Forum Consensus Conference [21]. However, despite this, there might be differences in the diagnosis of infection among different centers and researchers. Especially regarding VAP, it may not always be possible to differentiate between colonization and infection. These factors may have contributed to the lack of difference in mortality rates for the CR and CS strains in this study. Another explanation why carbapenem resistance was not found as a risk factor for mortality in this study could be the low percentage of bacteremia cases (9.5% among patients infected with *Acinetobacter* spp., *Klebsiella* spp., or *Pseudomonas* spp.). A significant difference in the death rates was not detected among patients with infections other than bacteremia, undetermined infections, or a low percentage of bacteremia cases [18, 19]. We did not report the appropriate empirical antibiotic treatment in this study, which is a limitation of the study. However, the likelihood of using inappropriate initial antibiotics in patients infected with CR strains is significantly higher than that of those infected with CS strains [46]. In the present study, the mortality rate was already similar between patients infected with CR and CS strains even without considering appropriate initial antibiotic treatment.

### Candida infections and mortality

Candida infections were found to be independent risk factors for mortality in this study. Although infection with *Acinetobacter* spp. was significantly higher in non-survivors than survivors, infection with *Acinetobacter* spp. was not an independent predictor of mortality after adjusting for confounders. Candida bloodstream infections, the majority of nosocomial fungal infections, are a significant cause of mortality in the ICU [47, 48]. According to data from the EUROBACT study [48], fungemia (82.6% are caused by *Candida* spp.) was associated with higher mortality. In the present study, 53.6% of Candida infections were bloodstream infections. There was a longer delay in initiation of antifungal treatment for fungemia than there was for antibiotics for bacteremia [48]. In the present study, appropriate initial antifungal treatment was not considered which could have contributed to the finding of Candida infections as an independent risk factor for mortality. Nevertheless, the results of studies investigating the benefit of early antifungal treatment in patients with candidemia are contradictory [48, 49].

### Septic shock according to the SEPSIS-III definition

This study was performed before the publication of the new sepsis definitions (SEPSIS-III). According to the SEPSIS-III definition, 104 (6.9%) patients were found to have septic shock. When septic shock was defined according to the SEPSIS-III definition, it was associated with a higher number of organ dysfunctions (4.4 ± 11.3 and 3.7 ± 1.3, respectively) and mortality rate (75.9% and 70.4%, respectively) in comparison to the 1992 septic shock definitions (SEPSIS-I). However, there are several limitations in applying the new SEPSIS-III definition of septic shock to data from this study. Our database was constructed according to the 1992 consensus criteria of the ACCP/SCCM (SEPSIS-1). Patients who had a mean arterial pressure between 65 and 70 mmHg and were not receiving vasopressors were classified as hypotensive in our database. However, these patients are not classified as having septic shock according to SEPSIS-III definitions. Therefore, the actual incidence of septic shock according to SEPSIS-III might be lower.

### Strengths and weakness of the study

This study has some limitations in addition to the lack of data regarding initial appropriate antibiotic treatment. First of all, because of study participation was on a voluntary basis and sampling of ICUs was not a random sample from Turkey, the results might be biased to include more severely ill patients. In Turkey, about 42% of all ICU beds belong to private hospitals. We observed that only a few ICUs of private hospitals participated in this study. Exclusion of private hospitals could have led to an overestimation of mortality because fewer severely ill patients are looked after in private hospitals. There are 15,513 ICU beds in Turkey, and 1605 patients consented to participate in this study. Therefore, nearly 90% of Turkey’s ICU population did not contribute to the study which is a limitation to generalizability of study results. Second, as mentioned above, it might not have been always possible to differentiate between colonization and infection, especially in terms of VAP cases. These factors may have contributed to the lack of difference in the mortality rates for the CR and CS strains in this study. Furthermore, the doses of antibiotics were not reported, which could be an important variable when the outcomes for patients with sepsis are compared. Third, the educational qualifications of the nurses were not considered in this study. Lastly, this was a point prevalence study, and the prevalence of disease is influenced by both the incidence and duration of disease. Given a constant incidence, an increase in fatality or improvement in disease treatment can decrease the prevalence rate. Despite these limitations, this was a prospective, large epidemiologic study, which provided valuable information about the epidemiology of sepsis in ICUs in Turkey, a middle-income country that is among the countries with a high antibiotic resistance level. To the best of our knowledge, this is the first study to show that the low nurse-to-patient ratio is associated with mortality in infected patients.

## Conclusions

A high prevalence of sepsis and an unacceptably high mortality rate were observed in Turkish ICUs. The carbapenem resistance rate was high in Turkish ICUs, but it was not associated with a higher risk for mortality. Age, APACHE II score at ICU admission, SOFA score on study day, solid organ malignancy, sepsis severity, *Candida* spp. infection, RRT, and a nurse-to-patient ratio of 1:4 (compared with a nurse-to-patient ratio of 1:2) were found to be independent predictors of mortality in infected patients.

## Additional files

Additional file 1: **Table S1.** Intraclass correlation coefficients (ICCs) with 95% CI for the association between predictor variables and mortality, and design effects (Deff) for the entire cohort of infected patients. (DOCX 26 kb)

Additional file 2: **Table S2.** Prevalence of infection, sepsis, severe sepsis, and septic shock by hospital and intensive care unit type. (DOCX 100 kb)

Additional file 3: **Table S3.** Monitoring techniques and therapies used in patients with severe sepsis and septic shock on study day. (DOCX 76 kb)

Additional file 4: **Table S4.** Identification of clinical and microbiologic variables associated with 30-day mortality in patients infected with *Acinetobacter*, *Klebsiella*, or *Pseudomonas* spp. using univariate analysis. (DOCX 85 kb)

Additional file 5: **Table S5.** Identification of clinical and microbiologic variables associated with 30-day mortality using univariate analysis in the entire cohort of infected patients. (DOCX 96 kb)

## Abbreviations

ACCP/SCCM: American College of Chest Physicians and Society of Critical Care Medicine
APACHE II: Acute physiology and chronic health evaluation II
CI: Confidence intervals
CR: Carbapenem-resistant
CRF: Case report form
CS: Carbapenem-sensitive
Deff: Design effect
EPIC II: International study of the prevalence and outcomes of infection intensive care units II
ICC: Intraclass correlation coefficient
ICU: Intensive care unit
MRSA: Methicillin-resistant *Staphylococcus aureus*
MV: Mechanical ventilation
RRT: Renal replacement therapy
SBP: Systolic blood pressure
SIRS: Systemic inflammatory response syndrome
SOFA: Sequential organ failure assessment
