# Supplementary material for: Epidemiology of sepsis in intensive care units in Turkey: a multicenter, point-prevalence study
Source: Crit Care. 2018 Apr 16;22:93. doi: 10.1186/s13054-018-2013-1 (PMC5901868; doi:10.1186/s13054-018-2013-1)
Supplement: Supplementary file 1 — Table S1. Intraclass correlation coefficients (ICCs) with 95% CI for the association between predictor variables and mortality, and design effects (Deff) for the entire cohort of infected patients. (DOCX 26 kb) [file 13054_2018_2013_MOESM1_ESM.docx]

**Table S1: Intraclass correlation coefficients (ICCs) with 95% CI for the association between predictor variables and mortality, and design effects (Deff) for the entire cohort of infected patients**

|  | Average cluster size  (hospitals) | Deff | ICC_h_(95% CI) | SE | Average cluster size**^b^**  (ICUs) | Deff | ICC_i_(95% CI) | SE |
| --- | --- | --- | --- | --- | --- | --- | --- | --- |
| **Type of hospital** | 9.1 | 1.32 | 0.04  (0.003-0.386) | 0.05 | 7.3 | 1.44 | 0.07  (0.030-0.173) | 0.03 |
| State hospital**^a^** |  |  |  |  |  |  |  |  |
| Education and research hospital |  |  |  |  |  |  |  |  |
| University hospital, |  |  |  |  |  |  |  |  |
| Private hospital |  |  |  |  |  |  |  |  |
| **Hospital size** | 9.1 | 1.24 | 0.03  (0.002-0.404) | 0.05 | 7.3 | 1.44 | 0.07  (0.002-0.431) | 0.03 |
| >600 beds^a^ |  |  |  |  |  |  |  |  |
| 401–600 beds |  |  |  |  |  |  |  |  |
| 201–400 beds |  |  |  |  |  |  |  |  |
| <200 beds |  |  |  |  |  |  |  |  |
| **Nurse to patient ratio** | 9.1 | 1.24 | 0.03  (002-0.431) | 0.05 | 7.3 | 1.44 | 0.07  (0.028-0.170) | 0.03 |
| ½^a^ |  |  |  |  |  |  |  |  |
| 1/3 |  |  |  |  |  |  |  |  |
| ¼ |  |  |  |  |  |  |  |  |
| **Age, yr** | 9.1 | 1.32 | 0.04  (0.004-0.323) | 0.05 | 7.3 | 1.58 | 0.08  (0.033-0.179) | 0.03 |
| **Gender(F/M)** | 9.1 | 1.32 | 0.04  (0.002-0.392) | 0.05 | 7.3 | 1.44 | 0.07  (0.029-0.172) | 0.03 |
| **APACHE II, per point** | 9.0 | 1.24 | 0.03  (0.001-0.492) | 0.05 | 7.2 | 1.68 | 0.11  (0.053-0.223) | 0.04 |
| **SOFA,per point** | 9.0 | 1.16 | 0.02  (0.0003-0.707) | 0.06 | 7.2 | 1.68 | 0.11  (0.05-0.228) | 0.04 |
| **Comorbid conditions** |  |  |  |  |  |  |  |  |
| Chronic respiratory failure | 9.1 | 1.32 | 0.04  (0.004-0.351) | 0.05 | 7.3 | 1.44 | 0.07  (0.028-0.171) | 0.03 |
| Cerebrovascular accident | 9.1 | 1.24 | 0.03  (0.002-0.407) | 0.05 | 7.3 | 1.44 | 0.07  (0.029-0.172) | 0.03 |
| Congestive heart failure | 9.1 | 1.32 | 0.04  (0.003-0.392) | 0.05 | 7.3 | 1.58 | 0.08  (0.035-0.184) | 0.03 |
| Chronic renal failure | 9.1 | 1.32 | 0.04  (0.005-0.314) | 0.05 | 7.3 | 1.44 | 0.07  (0.02-0.172) | 0.03 |
| ID-Diabetes mellitus | 9.1 | 1.32 | 0.04  (.003-0.382) | 0.05 | 7.3 | 1.44 | 0.07  (0.031-0.176) | 0.03 |
| Solid organ malignancy | 9.1 | 1.24 | 0.03  (0.001-0.420) | 0.05 | 7.3 | 1.44 | 0.07  (0.030-0.174) | 0.03 |
| Immunosuppression | 9.1 | 1.16 | 0.02  (0.0004-0.642) | 0.05 | 7.3 | 1.44 | 0.07  (0.028-0.170) | 0.03 |
| Chronic liver disease | 9.1 | 1.24 | 0.03  (0.0009-0.513) | 0.05 | 7.3 | 1.44 | 0.07  (0.029-0.172) | 0.03 |
| Alcoholism | 9.1 | 1.32 | 0.04  (0.003-0.380) | 0.05 | 7.3 | 1.44 | 0.07  (0.028-0.171) | 0.03 |
| **Admission category** | 9.1 | 1.24 | 0.03  (0.002-0.395) | 0.05 | 7.2 | 1.31 | 0.06  (0.02-0.172) | 0.03 |
| Trauma^a^ |  |  |  |  |  |  |  |  |
| Emergency surgery |  |  |  |  |  |  |  |  |
| Elective surgery |  |  |  |  |  |  |  |  |
| Medical |  |  |  |  |  |  |  |  |
| **Infection source** |  |  |  |  |  |  |  |  |
| Respiratory | 9.1 | 1.32 | 0.04  (0.003-0.364) | 0.05 | 7.3 | 1.44 | 0.07  (0.029-0.171) | 0.03 |
| Bloodstream | 9.1 | 1.32 | 0.04  (0.002-0.398) | 0.05 | 7.3 | 1.44 | 0.07  (0.031-0.176) | 0.03 |
| Renal/Urinary | 9.1 | 1.32 | 0.04  (0.003-0.390) | 0.05 | 7.3 | 1.44 | 0.07  (0.031-0.175) | 0.03 |
| Catheter-related | 9.1 | 1.32 | 0.04  (0.004-0.362) | 0.05 | 7.3 | 1.44 | 0.07  (0.031-0.176) | 0.03 |
| Intraabdominal | 9.1 | 1.32 | 0.04  (0.003-0.393) | 0.05 | 7.3 | 1.44 | 0.07  (0.031-0.176) | 0.03 |
| Surgical | 9.1 | 1.32 | 0.04  (0.003-0.385) | 0.05 | 7.3 | 1.44 | 0.07  (0.029-0.172) | 0.03 |
| Skin-soft tissue | 9.1 | 1.32 | 0.04  (0.003-0.377) | 0.05 | 7.3 | 1.44 | 0.07  (0.029-0.173) | 0.03 |
| **Organ dysfunction** |  |  |  |  |  |  |  |  |
| Respiratory | 9.1 | 1.40 | 0.05  (0.007-0.331) | 0.05 | 7.3 | 1.58 | 0.08  (0.033-0.188) | 0.03 |
| Renal | 9.1 | 1.16 | 0.02  (0.0005-0.582) | 0.05 | 7.3 | 1.44 | 0.07  (0.028-0.184) | 0.03 |
| Liver | 9.1 | 1.08 | 0.01  (0.00001-0.953) | 0.05 | 7.3 | 1.44 | 0.07  (0.031-0.175) | 0.03 |
| Acute encephalopathy | 9.1 | 1.40 | 0.05  (0.007-0.3189 | 0.05 | 7.3 | 1.50 | 0.08  (0.034-0.183) | 0.03 |
| Hematologic | 9.1 | 1.08 | 0.01  (.00003-0.920) | 0.06 | 7.3 | 1.37 | 0.06  (0.023-0.168) | 0.03 |
| **Lactic acidosis,**  **(>2 mmol∙L^-1^)** | 9.1 | 1.16 | 0.02  (0.00008-0.854) | 0.06 | 7.3 | 1.44 | 0.07  (0.027-0.175) | 0.03 |
| **Clinical condition** | 9.1 | 1.16 | 0.02  (0.00006-0.875) | 0.06 | 7.3 | 1.31 | 0.05  (0.013-0.182) | 0.03 |
| Infection^a^ |  |  |  |  |  |  |  |  |
| Infection+SIRS |  |  |  |  |  |  |  |  |
| Severe sepsis without shock |  |  |  |  |  |  |  |  |
| Septic shock |  |  |  |  |  |  |  |  |
| **Type of micro-organism** |  |  |  |  |  |  |  |  |
| *Acinetobacter* spp. | 9.1 | 1.32 | 0.04  (0.003-0.400) | 0.05 | 7.3 | 1.44 | 0.07  (0.031-0.175) | 0.03 |
| *Pseudomonas* spp. | 9.1 | 1.32 | 0.04  (0.004-0.377) | 0.05 | 7.3 | 1.44 | 0.07  (0.031-0.175) | 0.03 |
| *Klebsiella* spp. | 9.1 | 1.32 | 0.04  (0.002-0.391) | 0.05 | 7.3 | 1.44 | 0.07  (0.029-0.173) | 0.03 |
| *Staphylococcus aureus* | 9.1 | 1.32 | 0.04  (0.002-1.060) | 0.08 | 7.3 | 1.44 | 0.07  (0.029-0.172) | 0.03 |
| *Enterococcus* spp. | 9.1 | 1.32 | 0.04  (0.003-0.387) | 0.05 | 7.3 | 1.44 | 0.07  (0.030-0.173) | 0.03 |
| *Candida* spp. | 9.1 | 1.40 | 0.05  (0.007-0.323) | 0.05 | 7.3 | 1.44 | 0.07  (0.030-0.172) | 0.03 |
| *Escherichia coli* | 9.1 | 1.32 | 0.04  (0.004-0.355) | 0.05 | 7.3 | 1.44 | 0.07  (0.030-0.174) | 0.03 |
| *Serratia* spp. | 9.1 | 1.32 | 0.04  (0.003-0.387) | 0.05 | 7.3 | 1.44 | 0.07  (0.030-0.174) | 0.03 |
| *Proteus* spp. | 9.1 | 1.32 | 0.04  (0.002-0.387) | 0.05 | 7.3 | 1.44 | 0.07  (0.030-0.174) | 0.03 |
| *Enterobacter* spp. | 9.1 | 1.32 | 0.04  (0.002-0.390) | 0.05 | 7.3 | 1.44 | 0.07  (0.029-0.172) | 0.03 |
| **Polymicrobial infection** | 9.1 | 1.24 | 0.03  (0.002-0.390) | 0.05 | 7.3 | 1.44 | 0.07  (0.027-0.169) | 0.03 |
| **Multiple infection** | 9.1 | 1.32 | 0.04  (0.004-0.358) | 0.05 | 7.3 | 1.44 | 0.07  (0.031-0.175) | 0.03 |
| **Therapies, n(%)** |  |  |  |  |  |  |  |  |
| MV | 9.1 | 1.32 | 0.04  (0.001-0.533) | 0.07 | 7.3 | 1.63 | 0.10  (0.049-0.209) | 0.03 |
| RRT | 9.1 | 1.16 | 0.02  (0.0006-0.540) | 0.05 | 7.3 | 1.31 | 0.05  (0.015-0.161) | 0.03 |

**ICCh,** the level-3 intraclass correlation coefficient at the hospital level.

**ICC_i,_** the level -2 Intraclass correlation coefficient for the ICU within hospital level.

**SE,** standard errors.

**^a^**Reference category.

**SIRS,** systemic inflammatory response syndrome; **APACHE II,** acute physiology and chronic health evaluation; **ICU,** intensive care unit, **SOFA,** sequential organ failure assessment; **MV,** Mechanical ventilation; **RRT,** renal replacement treatment.

**^b^**1499 patients from 132 ICUs within 94 hospitals were included to the study to identify infection ratio in ICUs in Turkey. But, only patients with an infection (n=863) were screened for the presence of sepsis, and an extended documentation including survival status were performed in only infected patients. Because some intensive care units did not have an infected patient on study day and thus 863 infected patients were from 119 ICUs within 94 hospitals.
