# Supplementary material for: Epidemiology of sepsis in intensive care units in Turkey: a multicenter, point-prevalence study
Source: Crit Care. 2018 Apr 16;22:93. doi: 10.1186/s13054-018-2013-1 (PMC5901868; doi:10.1186/s13054-018-2013-1)
Supplement: Supplementary file 2 — Table S2. Prevalence of infection, sepsis, severe sepsis, and septic shock by hospital and intensive care unit type. (DOCX 100 kb) [file 13054_2018_2013_MOESM2_ESM.docx]

**Table S2: Prevalence of infection, sepsis, severe sepsis, and septic shock by hospital and intensive care unit type**

|  | **Distribution of infected patients** | | | | | |
| --- | --- | --- | --- | --- | --- | --- |
|  | **No infection**  **n=636** | **Totally infected**  **n=863** | **Infection**  **n=237** | **Infection+SIRS**  **n=163** | **Severe sepsis without shock**  **n=260** | **Septic shock**  **n=203** |
| **Type of hospital, n (%)** | | | | | | |
| State hospital | **49 (37.4)** | **82 (62.6)** | 21 (16.0) | 18 (13.7) | 25 (19.0) | 18 (13.7) |
| Education and research hospital | **229 (42.0)** | **316 (57.9)** | 88 (16.1) | 56 (10.3) | 111 (20.4) | 60 (11.0) |
| University hospital | **331 (42.8)** | **442 (57.2)** | 120 (15.5) | 86 (11.1) | 117 (15.1) | 115 (14.8) |
| Private hospital | **23 (46.0)** | **27 (54.0)** | 7 (14.0) | 3 (6.0) | 6 (12.0) | 11 (22.0) |
| **Size of hospital, n (%)** |  |  |  |  |  |  |
| >600 beds | **340 (44.5)** | **424 (55.5)** | 112 (14.6) | 78 (10.2) | 130 (17.0) | 102 (13.4) |
| 401–600 beds | **136 (40.1)** | **203 (59.9)** | 43 (12.7) | 48 (14.2) | 69 (20.4) | 42 (12.4) |
| 201–400 beds | **131 (42.8)** | **175 (57.2)** | 63 (20.6) | 27 (8.8) | 42 (13.7) | 43 (14.0) |
| <200 beds | **25 (27.8)** | **65 (72.2)^*^** | 19 (21.1) | 10 (11.1) | 19 (21.1) | 17 (17.7) |
| **Type of ICU, n (%)** |  |  |  |  |  |  |
| Mixed medical/surgical | **495 (41.7)** | **693 (58.3)** | 184 (15.5) | 131 (11.0) | 222 (18.7) | 156 (13.1) |
| Surgical | **51 (43.6)** | **66 (56.4)** | 16 (13.7) | 15 (12.8) | 20 (17.1) | 15 (12.8) |
| Medical | **79 (46.7)** | **90 (53.3)** | 31 (18.3) | 14 (8.3) | 16 (9.5) | 29 (17.2) |
| Neurological | **11 (44.0)** | **14 (56.0)** | 6 (24.0) | 3 (12.0) | 2 (8.0) | 3 (12.0) |

^*^p<0.05, ratio of infected patients were significantly higher in hospitals with a bed capacity <200 compared to that with a bed capacity of 201–400, 401–600, or >600.

**SIRS,** systemic inflammatory response syndrome; **ICU,** intensive care unit.
