# Supplementary material for: Epidemiology of sepsis in intensive care units in Turkey: a multicenter, point-prevalence study
Source: Crit Care. 2018 Apr 16;22:93. doi: 10.1186/s13054-018-2013-1 (PMC5901868; doi:10.1186/s13054-018-2013-1)
Supplement: Supplementary file 3 — Table S3. Monitoring techniques and therapies used in patients with severe sepsis and septic shock on study day. (DOCX 76 kb) [file 13054_2018_2013_MOESM3_ESM.docx]

**Table S3: Monitoring techniques and therapies used in patients (N=463) with severe sepsis and septic shock on study day.**

| **Monitoring technique** | **n (%)** |
| --- | --- |
| Measurement of CVP | 341 (73.6) |
| Measurement of ScvO_2_ | 89 (19.2) |
| Lactate measurement | 370 (79.9) |
| Measurement of hourly urine output | 427 (92.2) |
| Invasive blood pressure measurement | 274 (59.1) |
| **Therapies** |  |
| MV | 404 (87.2) |
| RRT | 110 (23.7) |
| Corticosteroid | 124 (26.7) |
| Inotropic/vasopressor agent | 227 (49) |
| Extracorporeal cytokine removal | 16 (3.4) |
| ECMO | 7 (1.5) |
| IVIG | 4 (0.8) |
| **Nutrition** |  |
| Enteral | 270 (58.3) |
| Parenteral | 93 (20) |
| Enteral and parenteral | 68 (14.6) |
| No nutrition | 18 (3.8) |

**CVP,** central venous pressure; **ScvO2,** central venous oxygen saturation; **MV,** mechanical ventilation; **RRT,** renal replacement therapy; **ECMO,** extracorporeal membrane oxygenation; **IVIG,** intravenous immunoglobulin.
