# Supplementary material for: Epidemiology of sepsis in intensive care units in Turkey: a multicenter, point-prevalence study
Source: Crit Care. 2018 Apr 16;22:93. doi: 10.1186/s13054-018-2013-1 (PMC5901868; doi:10.1186/s13054-018-2013-1)
Supplement: Supplementary file 4 — Table S4. Identification of clinical and microbiologic variables associated with 30-day mortality in patients infected with Acinetobacter, Klebsiella, or Pseudomonas spp. using univariate analysis. (DOCX 85 kb) [file 13054_2018_2013_MOESM4_ESM.docx]

**Table S4: Identification of clinical and microbiologic variables associated with 30-days mortality in patients infected with *Acinetobacter*, *Klebsiella*, or *Pseudomonas* spp.** **using univariate analysis**

|  | **Survivors**  **(n=166)** | **Non-survivors**  **(n=192)** | **Univariate**  **OR** | **95% CI** | **P-value** |
| --- | --- | --- | --- | --- | --- |
| **Age, yrs*** | 64 (47–76) | 71 (60–80) | 1.02 | 1.013–1.038 | <0.001 |
| **Female/Male, n** | 69/97 | 77/115 | 1.09 | 0.714-1.667 | 0.688 |
| **APACHE II score at admission ^b,*^** | 21 (16–27) | 23 (18–31) | 1.02 | 1.005–1.045 | 0.016 |
| **SOFA score on study day  ^b,*^** | 7 (4–9) | 8.5 (6–11.3) | 1.13 | 1.064–1.197 | <0.001 |
| **Comorbid conditions, n (%)** |  |  |  |  |  |
| Chronic respiratory failure | 43 (25.9) | 49 (25.5) | 0.99 | 0.618–1.599 | 0.980 |
| Cerebrovascular accident | 35 (21.1) | 33 (17.2) | 0.78 | 0.457–1.317 | 0.347 |
| Congestive heart failure* | 11 (6.6) | 29 (15.1) | 2.52 | 1.218–5.225 | 0.013 |
| Chronic renal failure* | 11 (6.6) | 23 (11.9) | 1.93 | 0.911–4.089 | 0.086 |
| ID-Diabetes mellitus | 12(7.2) | 19(10) | 1.42 | 0.670–3.033 | 0.357 |
| Solid organ malignancy* | 13 (7.8) | 24 (12.5) | 1.69 | 0.832–3.440 | 0.146 |
| Immunosuppression* | 7 (4.2) | 18 (9.4) | 2.36 | 0.962–5.810 | 0.061 |
| Chronic liver disease | 2 (1.2) | 3 (1.6) | 1.31 | 0.216–7.933 | 0.769 |
| Alcoholism | 5 (3.0) | 7 (3.6) | 1.23 | 0.383–3.957 | 0.726 |
| **Clinical condition, n (%)*** |  |  |  |  | <0.001 |
| Infection **^a^** | 61 (36.7) | 28 (14.6) | 1 |  |  |
| Infection+SIRS | 34 (20.5) | 26 (13.5) | 1.66 | 0.845–3.285 | 0.141 |
| Severe sepsis without shock | 46(27.7) | 58 (30.2) | 2.74 | 1.520–4.963 | 0.001 |
| Septic shock | 25 (15.1) | 78 (40.6) | 6.79 | 3.602–12.825 | <0.001 |
| **Type of infection, n (%)** |  |  |  |  |  |
| Respiratory | 132 (79.5) | 148 (77.0) | 0.87 | 0.529–1.440 | 0.594 |
| Bloodstream | 14 (8.4) | 20 (10.4) | 1.26 | 0.616–2.586 | 0.524 |
| Renal/urinary | 5 (3.0) | 5 (2.6) | 0.86 | 0.245–3.027 | 0.815 |
| Catheter related | 11 (6.6) | 10 (5.2) | 0.96 | 0.380–2.418 | 0.928 |
| Skin/soft tissue | 4 (2.4) | 3 (1.6) | 0.64 | 0.142–2.915 | 0.567 |
| Wound | 5 (3.0) | 5 (2.6) | 0.68 | 0.181–2.594 | 0.578 |
| Intraabdominal | 4 (2.4) | 5 (2.6) | 1.08 | 0.286–4.101 | 0.907 |
| Others | 1 (0.6) | 2 (1.0) | 1.74 | 0.156–19.328 | 0.653 |
| **Polymicrobial infection*** | 21 (12.7) | 27 (14.1) | 1.13 | 0.612–2.084 | 0.696 |
| **Multiple infection*** | 16 (9.6) | 16 (8.3) | 0.85 | 0.412–1.762 | 0.666 |
| **Carbapenem resistant*** | 95 (56.9) | 112 (58.3) | 1.06 | 0.706–1.636 | 0.738 |
| **Type of micro-organism, n (%)** |  |  |  |  |  |
| *Acinetobacter* spp.* | 96 (57.8) | 113 (58.9) | 1.05 | 0.695–1.611 | 0.793 |
| *Pseudomonas* spp.* | 40 (24.1) | 52 (27.1) | 1.17 | 0.732–1.900 | 0.498 |
| *Klebsiella* spp.* | 45 (27.1) | 46 (23.9) | 0.85 | 0.531–1.375 | 0.516 |
| **Therapies, n (%)** |  |  |  |  |  |
| MV* | 147 (88.5) | 177 (92.2) | 1.76 | 0.841–3.683 | 0.134 |
| RRT* | 24 (14.6) | 55 (28.6) | 2.39 | 1.403–4.079 | 0.001 |
| **Length of stay prior to study day, days*** | 19 (9–36) | 14.5 (6–29) | 1 | 0.998–1.002 | 0.866 |

Data are presented as median (25^th^–75^th^ percentiles), if not otherwise specified.

**^a^** Reference category.

**^b^** 5 missing values.

* Variables included into multiple logistic regression analysis.

**OR,** odds ratio; CI, confidence interval; **APACHE II,** acute physiology and chronic health evaluation II; **SOFA,** sequential organ failure assessment; **SIRS,** systemic inflammatory response syndrome; **MV**, mechanical ventilation; **RRT,** renal replacement therapy; **ID-diabetes mellitus,** insulin-dependent diabetes mellitus.
