# Supplementary material for: Epidemiology of sepsis in intensive care units in Turkey: a multicenter, point-prevalence study
Source: Crit Care. 2018 Apr 16;22:93. doi: 10.1186/s13054-018-2013-1 (PMC5901868; doi:10.1186/s13054-018-2013-1)
Supplement: Supplementary file 5 — Table S5. Identification of clinical and microbiologic variables associated with 30-day mortality using univariate analysis in the entire cohort of infected patients. (DOCX 96 kb) [file 13054_2018_2013_MOESM5_ESM.docx]

**Table S5. Identification of clinical and microbiologic variables associated with 30-days mortality in the entire cohort of infected patients using univariate analysis**

|  | **Survivors**  **(n=462)** | **Non-survivors**  **(n=401)** | **Univariate**  **OR** | | **95% CI** | **P-value** |
| --- | --- | --- | --- | --- | --- | --- |
| **Type of hospital, n (%)** |  |  |  |  | | **0.945** |
| State hospital**^a^** | 43 (9.3) | 41 (10.2) | 1 |  | |  |
| Education and research hospital | 169 (36.6) | 146 (36.4) | 0.90 | 0.560–1.467 | |  |
| University hospital | 237 (51.3) | 201 (50.1) | 0.89 | 0.557–1.419) | |  |
| Private hospital | 13 (2.8) | 13 (3.2) | 1.05 | 0.435–2.528 | |  |
| **Hospital size** |  |  |  |  | | **0.207** |
| >600 beds, n (%) | 227 (49.1) | 195 (48.6) | 1 |  | |  |
| 401–600 beds | 116 (25.1) | 86 (21.4) | 0.65 | 0.369–1.174 | |  |
| 201–400 beds | 92 (19.9) | 83 (20.7) | 0.54 | 0.306–0.956 | |  |
| <200 beds | 27 (5.8) | 37 (9.2) | 0.63 | 0.368–1.067 | |  |
| **Nurse to patient ratio, n (%)*** |  |  |  |  | | **0.130** |
| 1:2**^a^** | 180 (38.5) | 131 (32.4) | 1 |  | |  |
| 1:3 | 199 (43.0) | 187 (46.6) | 1.29 | 0.956–1.744 | |  |
| 1:4 | 81 (17.5) | 83 (20.7) | 1.40 | 0.963–2.059 | |  |
| **Age, yrs*** | 66 (50.7–77) | 72 (59–81) | 1.02 | 1.012–1.029 | | **<0.001** |
| **APACHE II score at**  **admission ^c, *^** | 20 (15–26) | 23 (18–30) | 1.03 | 1.023–1.051 | | **<0.001** |
| **SOFA score on study day ^c, *^** | 6 (4–9) | 9 (6–13) | 1.18 | 1.138–1.225 | | **<0.001** |
| **Female/male, n** | 200/262 | 166/235 | 1.08 | 0.824–1.417 | | **0.575** |
| **Comorbid conditions, n (%)** |  |  |  |  | |  |
| Chronic respiratory failure | 100 (21.6) | 92 (22.9) | 1.07 | 0.779–1.482 | | **0.660** |
| Cerebrovascular accident | 65 (14) | 53 (13.2) | 0.93 | 0.628–1.371 | | **0.707** |
| Congestive heart failure* | 37 (8.0) | 61 (15.2) | 2.39 | 1.540–3.711 | | **<0.001** |
| Chronic renal failure* | 34 (7.4) | 61 (15.2) | 2.26 | 1.450–3.517 | | **<0.001** |
| ID-Diabetes mellitus | 40 (8.7) | 44 (10.9) | 1.3 | 0.828–2041 | | **0.254** |
| Solid organ malignancy * | 33 (7.2) | 48 (11.9) | 1.77 | 1.110–2.814 | | **0.016** |
| Immunosuppression* | 24 (5.2) | 43 (10.7) | 2.04 | 1.223–3.421 | | **0.006** |
| Chronic liver disease* | 7 (1.5) | 15 (3.7) | 2.52 | 1.019–6.259 | | **0.045** |
| Alcoholism | 8 (1.7) | 9 (2.2) | 1.30 | 0.499–3.418 | | **0.773** |
| **Admission category, n (%)^c, *^** |  |  |  |  | | **0.119** |
| Trauma **^a^** | 35 (7.6) | 15 (3.8) | 1 |  | |  |
| Emergency surgery | 48 (10.5) | 39 (9.8) | 1.89 | 0.906–3.965 | |  |
| Elective surgery | 45 (9.8) | 43 (10.8) | 2.23 | 1.069–4.651 | |  |
| Medical | 330 (72.1) | 300 (75.6) | 2.12 | 1.136–3.962 | |  |
| **Infection source, n (%)** |  |  |  |  | |  |
| Respiratory | 326 (70.5) | 292 (72.8) | 1.19 | 0.889–1.613 | | **0.236** |
| Bloodstream* | 48 (10.3) | 29 (7.2) | 0.67 | 0.415–1.089 | | **0.111** |
| Renal/urinary | 38 (7.5) | 29 (7.2) | 0.87 | 0.526–1.44 | | **0.587** |
| Catheter-related  bloodstream infection | 29 (6.2) | 27 (6.7) | 1.08 | 0.627–1.854 | | **0.786** |
| Intraabdominal | 24 (5.1) | 25 (6.2) | 1.11 | 0.625–1.981 | | **0.717** |
| Surgical | 18 (3.8) | 14 (3.4) | 0.89 | 0.438–1.818 | | **0.754** |
| Skin-soft tissue | 12 (2.5) | 12 (2.9) | 1.37 | 0.608–3.101 | | **0.445** |
| Other | 13 (2.8) | 9 (2.2) | 0.79 | 0.337–1.884 | | **0.602** |
| **Organ dysfunction, n (%)** |  |  |  |  | |  |
| Respiratory* | 141(30.5) | 200(49.8) | 2.22 | 1.681–2.932 | | **<0.001** |
| Renal* | 86 (18.6) | 179 (44.6) | 3.49 | 2.579–4.740 | | **<0.001** |
| Liver* | 61 (13.2) | 93 (23.1) | 1.94 | 1.367–2.776 | | **<0.001** |
| Acute encephalopathy* | 98 (21.2) | 129 (32.1) | 1.83 | 1.351–2.498 | | **<0.001** |
| Hematologic* | 39 (8.4) | 92 (22.9) | 3.09 | 2.084–4.599 | | **<0.001** |
| **Lactic acidosis, n (%)***  **(>2 mmol∙L^-1^)** | 62 (13.4) | 120 (29.9) | 2.66 | 1.893–3.734 | | **<0.001** |
| **Clinical condition, n (%)*** |  |  |  |  | | **<0.001** |
| Infection ^a^ | 176 (38.0) | 59 (14.7) | 1 |  | |  |
| Infection+SIRS | 110 (23.8) | 51 (12.7) | 1.35 | 0.871–2.098 | |  |
| Severe sepsis without shock | 115 (24.8) | 145 (36.1) | 3.63 | 2.487–5.322 | |  |
| Septic shock | 58 (12.5) | 142 (35.4) | 6.87 | 4.522–10.457 | |  |
| **Type of micro-organism, n (%)** |  |  |  |  | |  |
| *Acinetobacter* spp.* | 95 (20.3) | 113 (27.6) | 1.51 | 1.108–2.074 | | **0.009** |
| *Pseudomonas* spp.* | 40 (8.8) | 52 (12.7) | 1.57 | 1.017–2.431 | | **0.042** |
| *Klebsiella* spp. | 45 (9.7) | 44 (10.9) | 1.14 | 0.736–1.771 | | **0.553** |
| *Staphylococcus aureus* | 35 (7.5) | 23 (5.7) | 0.73 | 0.420–1.271 | | **0.267** |
| *Enterococcus* spp. | 19 (4.1) | 16 (3.9) | 0.97 | 0.491–1.911 | | **0.927** |
| *Candida* spp.* | 10 (2.1) | 21 (5.2) | 2.49 | 1.162–5.370 | | **0.019** |
| *Escherichia coli* | 20 (4.3) | 13 (3.2) | 0.74 | 0.364–1.512 | | **0.412** |
| *Serratia* spp. | 6 (1.2) | 5 (1.2) | 0.96 | 0.291–3.168 | | **0.946** |
| *Proteus* spp. | 4 (0.8) | 6 (1.5) | 1.74 | 0.487–6.207 | | **0.394** |
| *Enterobacter* spp. | 4 (0.8) | 4 (0.9) | 1.15 | 0.288–4.665 | | **0.835** |
| Gram-negative, others | 13 (2.8) | 8 (1.9) | 0.66 | 0.261–1.717 | | **0.404** |
| Gram-positive, cocci, others | 2 (0.4) | 4 (0.9) | 2.32 | 0.424–12.779 | | **0.331** |
| Virus (H1N1) | 5 (1.0) | 1 (0.2) | 0.23 | 0.032–2.584 | | **0.266** |
| **Polymicrobial infection*** | 25 (5.4) | 37 (9.2) | 1.77 | 1.050–3.007 | | **0.032** |
| **Multiple infection*** | 51 (11.0) | 52(12.9) | 1.20 | 0.795–1.812 | | **0.384** |
| **Therapies, n (%)** |  |  |  |  | |  |
| MV* | 358 (77.4) | 356 (88.7) | 2.29 | 1.573–3.358 | | **<0.001** |
| RRT* | 44 (9.5) | 108 (27) | 3.52 | 2.406–5.165 | | **<0.001** |

Data are presented as median (25^th^–75^th^ percentiles), if not otherwise specified.

**^a^** Reference category.

**^c^**8 missing values

* Variables included into multiple logistic regression analysis.

**OR,** odds ratio; **CI,** confidence interval; **APACHE II,** acute physiology and chronic health evaluation II; **SOFA,** sequential organ failure assessment; **SIRS,** systemic inflammatory response syndrome; **MV,** mechanical ventilation; **RRT,** renal replacement therapy; **ID-diabetes mellitus,** insulin- dependent diabetes mellitus.
